# Castelman's disease of the neck: a case report and literature review

**DOI:** 10.11604/pamj.2020.37.369.26909

**Published:** 2020-12-22

**Authors:** Amel El Korbi, Sondes Jellali, Mahbouba Jguiri, Ahlem Bellalah, Mehdi Ferjaoui, Rachida Bouatay, Naourez Kolsi, Khaled Harrathi, Jamel Koubaa

**Affiliations:** 1 ENT Department of Fattouma Bourguiba, University Hospital of Monastir, Monastir, Tunisia,; 2 Research Unit “Quality and Security of care” (UR12SP41), University of Monastir, Monastir, Tunisia,; 3 Department of Rheumatology, Fattouma Bourguiba University Hospital of Monastir, Monastir, Tunisia,; 4 Anatomical and Cytological Pathology Department, Fattouma Bourguiba University Hospital of Monastir, Monastir, Tunisia

**Keywords:** Castleman’s disease, lymphadenopathy, surgery

## Abstract

Castleman’s disease is a rare pathology, poorly understood. It is considered as a lymphoproliferative disorder, described for the first time in 1954, which may be confused with other causes of lymphadenopathy. We report in this paper the case of a young women presenting with left latero-cervical lymphadenopathy. All the investigations were negative except a large high-vascularized level II cervical lymphadenopathy. We performed a cervicotomy. The extemporaneous histological exam was non-contributive. We decided to perform a complete level II and III left cervical lymphadenectomy. The diagnosis of unicentric Castleman's disease was confirmed based on the final histological study of the specimen, and the absence of other cervical and extra-cervical lymphadenopathy. The patient is free of recurrence at the time of reporting this article.

## Introduction

Castleman´s disease (CD) is a rare, poorly understood lymphoproliferative disorder that share common lymph node histological features. The disease was first described in a single case in 954 [1], followed by a small cases series in 1956 [2]. Two clinical entities have been described: Unicentric with a confined disease to a single anatomic lymph node and multicentric characterized by generalized lymphadenopathy and more aggressive clinical course [3]. It has three histological subtypes: hyaline vascular, plasma cellular and mixed type. Most of the previously reported cases of CD in the neck were of the hyaline vascular type and the most common sign was an asymptomatic neck mass [4]. The aim of this paper is to describe a case of cervical unicentric CD, its diagnostic tools and the perioperative management.

## Patient and observation

**Figure 1:**
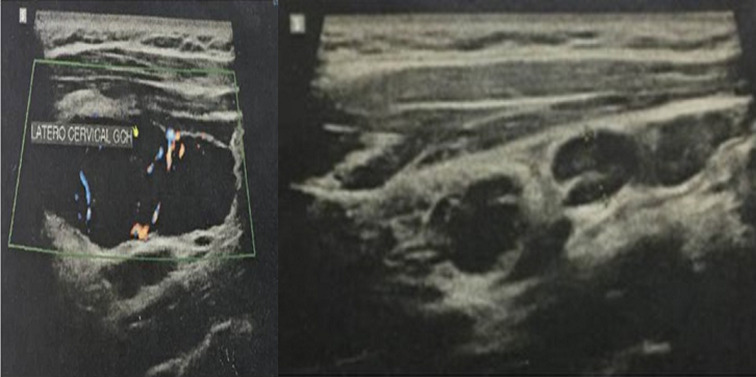
ultrasound images of cervical lymph nodes located in left IIB and V levels

**Figure 2:**
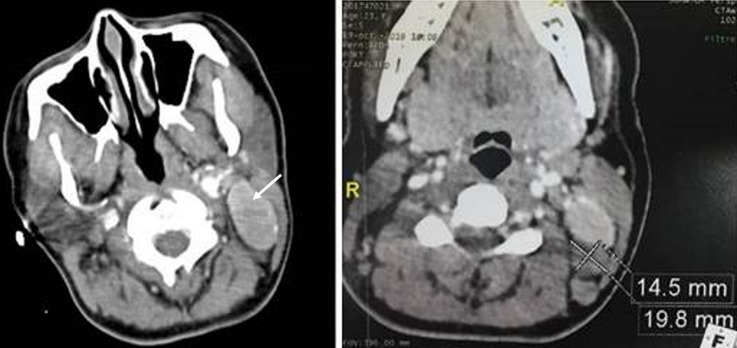
cervical CT scan with contrast enhancement showing cervical lymph node in left IIB and V levels with a strong enhancement of the IIB level lymph node (white arrow)

**Figure 3:**
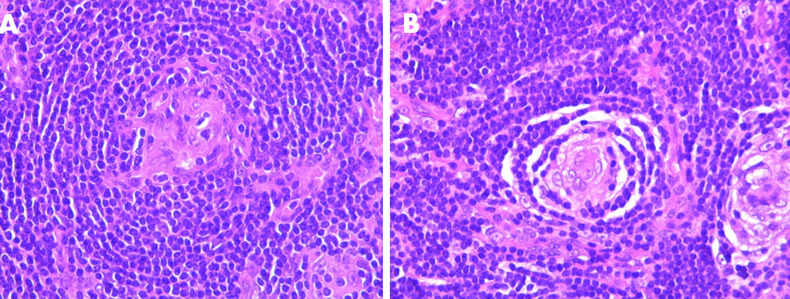
A) (HE x 100) the follicle is surrounded by a broad mantle zone consisting of a concentric layering of lymphocytes resulting in an “onion-skin” appearance; B) (HE x 400). The follicle is penetrated radially by a sclerotic blood vessel

A 23-year-old woman who presented with an isolated left side painless mass of the neck evolving for 8 months. The physical examination found multiple firm and non-fixed left lateral neck masses; the largest one measured five centimeters. Ultrasound echography showed a 4 centimeters well-limited hypoechoic oblong mass with central and peripheral vascularization of the level IIb with multiple centimeter lymph nodes at level V (Figure 1). Computed tomography (CT) showed multiple lymphadenopathies of the left levels II and V with an important enhancement after contrast sized between 20 and 41 millimeters in their largest dimension (Figure 2). Two fine needle aspirations attempts were non-contributive. A surgical biopsy under general anesthesia was carried but the extemporaneous examination was non-conclusive. We decided to perform a II, III and VA left levels lymphadenectomy removing the whole lymph nodes. The extirpation was laborious because of bleeding. The diagnosis of hyaline Castleman´s disease was made based on final histological findings (Figure 3). The thoraco-abdominal CT scan did not show any other lymph node considering though the unicentric form of CD. We did not observe recurrence or new lymphadenopathy appearance 12 months after surgery.

## Discussion

CD is a rare lymphoproliferative disorder, described for the first time by Castleman *et al*. [1,2] in 1954 in a single case then in 1956 in a group of 13 patients with localized benign lymphadenopathy. This entity is also termed localized nodal hyperplasia, angiomatous lymphoid hamartoma or giant lymph node hyperplasia [1]. Williams and Kaude reported that lymph nodes in the mediastinum were the most common (70%) and that the neck and abdomen were the least common (15%) sites for CD [5]. Although the exact pathogenesis of CD is unclear, studies have indicated that the occurrence of CD may be related to the infection of human herpes virus-8 (HHV-8) or human immunodeficiency virus (HIV), immune dysfunction and overproduction of interleukin-6 (IL-6). Asao *et al*. have proved IL-6 transgenic mice showed similar disorders related to CD, which suggested the contribution of IL-6 to CD [6]. The definitive diagnosis of CD is based on histopathological examination. Three types of CD have been identified: hyaline-vascular type, plasma cell type and mixed variant type [4,7]. Hyaline-vascular type is the most common clinical variant (90%) characterized by follicular hyperplasia with regressed germinal centers and important vascular proliferation. Plasma cell type is characterized by Russell bodies and exhibits larger lymphoreticular nodules and fewer hyalinized blood vessels compared with hyaline-vascular type. Mixed variant type is a rare variant and exhibits features of hyaline-vascular and plasma cell type [4].

The incidence of CD is unknown and can occur at any age, though it is mostly reported in adults in the literature with a slight feminine predominance (60%). There are two different clinical entities: the unicentric and the multicentric type. The localized form of the disease is mostly asymptomatic with a single site lymph node enlargement. Although localized CD most often occurs in the mediastinum, it may occur in any area of the body where lymph nodes are found such as the lung, neck, axilla, mesentery, pelvis, and retroperitoneum [6]. It is often discovered incidentally during routine examination, chest X rays or due to discomfort secondary to local compression. Diagnosis is made by histological analysis of the lymph node biopsy [7,8]. Multicentric form, however, presents with systemic symptoms along with multiple lymph node hyperplasia. The systemic symptoms are thought to be primarily a consequence of elevated Interleukin-6 (IL-6) production. They present as asthenia, weight loss, fever, polyadenopathy with a mean of four-site involvement and is often associated with hepatosplenomegaly [8]. Some forms of multicentric CD are associated with Kaposi´s sarcoma, which develops in the clinical course of most HIV positive multicentric CD cases. An association with HIV negative Kaposi´s sarcoma has also been seen to a much lesser extent [9]. HIV positive multicentric CD shows an increased prevalence of pulmonary symptoms and can be differentiated from other types of HIV-associated systemic lymphoproliferative disorders [9,10]. CD often shows well defined, mildly hypodense or isodense, homogeneous nodules or masses on non-enhanced computed tomography/magnetic resonance (CT/MR) images, and intermediate and marked enhancement on contrast-enhanced CT/MR images. The hypertrophied vessels are valuable features [11]. The calcification in affected lesions is not rare and is more commonly observed in hyaline vascular variant unicentric CD [12].

In our case, ultrasound and CT scan evoked the tuberculosis origin as a first diagnosis. Since cytological appearances vary depending on the type and extent of hyperplasia, fine needle aspiration cytology (FNAC) findings may not always be conclusive in all cases [13]. Even in our case, the two FNAC performed were non-contributive. Surgical resection is considered the cornerstone of curative treatment for unicentric CD and is the most widely accepted therapy in the literature. A systematic review by Talat *et al*. [14] of 278 unicentric patients found that surgical resection resulted in 95% disease-free survival at 3 years. If surgical removal is not possible, in cases that the lymph node is difficult to get to, radiation therapy may be an effective way to destroy the affected tissue. Radiation doses of 30-45 Gy appear to be effective, although tumor responses have been documented at lower doses [7]. Multicentric CD however tends to have a variable prognosis with no documented treatment consensus. A variety of combination treatments have been tried with surgical excision, chemotherapy and steroids [11]. In patients with associated Kaposi´s sarcoma monthly polychemotherapy (e.g. cyclophosphamide, vincristine, doxorubicin and prednisone) has been tried with limited success [8]. Anti-IL 6 antibodies have shown success with systemic symptoms, as have steroids. Most treatment modalities involve immunosuppression, increasing the chances of opportunistic infections [11]. Recent suggestions are that treatment with the anti-herpes virus drug gangciclovir or the antiCD20 B cell monoclonal antibody, rituximab, may markedly improve outcome [8].

## Conclusion

This case report is presented for its rarity. Neck lymph nodes are involved by CD and may be confused with other common causes of neck lymphadenopathy like tuberculosis and nodal secondaries. Surgical removal of the tumors in the unicentric type of CD is the treatment of choice. It is important to remember that all patients diagnosed with CD should receive a systemic survey to exclude the possibility of ignored lesions.
